# Perioperative Predictors of Right Ventricular Failure Following Left Ventricular Assist Device Implantation

**DOI:** 10.1097/MAT.0000000000002631

**Published:** 2025-12-23

**Authors:** Shruti Rajendra, Yousuf Salmasi, Binu Raj, Owais Dar, Nandor Marczin, Darrel Francis, Espeed Khoshbin

**Affiliations:** From the * National Heart & Lung Institute, Imperial College, London, UK; † Cardiothoracic Surgery, Harefield Hospital, Royal Brompton and Harefield as part of Guy’s and St Thomas NHS Trust, London, UK; ‡ Transplantation, Harefield Hospital, Royal Brompton and Harefield as part of Guy’s and St Thomas NHS Trust, London, UK; § Cardiology, Harefield Hospital, Royal Brompton and Harefield as part of Guy’s and St Thomas NHS Trust, London, UK; ¶ Cardiac Anaesthesiology, Harefield Hospital, Royal Brompton and Harefield as part of Guy’s and St Thomas NHS Trust, London, UK; ∥ Cardiothoracic Surgery, Harefield Hospital, Royal Brompton and Harefield as part of Guy’s and St Thomas NHS Trust, London, UK.

**Keywords:** left ventricular assist device, right ventricular failure

## Abstract

Right ventricular failure (RVF) is an important complication following implantation of a left ventricular assist device (LVAD) in patients with advanced heart failure. This study aims to identify perioperative hemodynamic predictors of RVF following LVAD implantation and develop an internally validated predictive model. Patients who underwent LVAD implantations between March 2013 and March 2023 at a large-volume tertiary center were retrospectively analyzed. The primary outcome was early post-implant RVF, defined by need for right ventricular assist device (RVAD), prolonged inotrope dependence (> 14 days), or death within 14 days while on inotropes. Perioperative hemodynamic variables were analyzed using univariate logistic regression. A prediction model was then developed using stepwise multivariate logistic regression, and internally validated using *k*-fold cross-validation. Among 210 patients, 73 patients (34.8%) developed early post-implant RVF. Decreased post-implant cardiac index (odds ratio [OR]: 0.0122, *p* = 0.001), decreased mean arterial pressure (OR: 0.932, *p* = 0.008), and simultaneously increased cardiac index and pulmonary pulse pressure (OR: 1.13, *p* = 0.021) independently predicted RVF. Preimplantation IV inotrope therapy was a significant confounder (OR: 3.96, *p* = 0.021). Our proposed predictive model achieved strong discriminatory power (area under the curve [AUC] of 0.830) and stable cross-validation performance. External validation with multicenter cohorts is warranted to confirm predictive accuracy and clinical applicability.

## Graphical abstract

**Figure:**
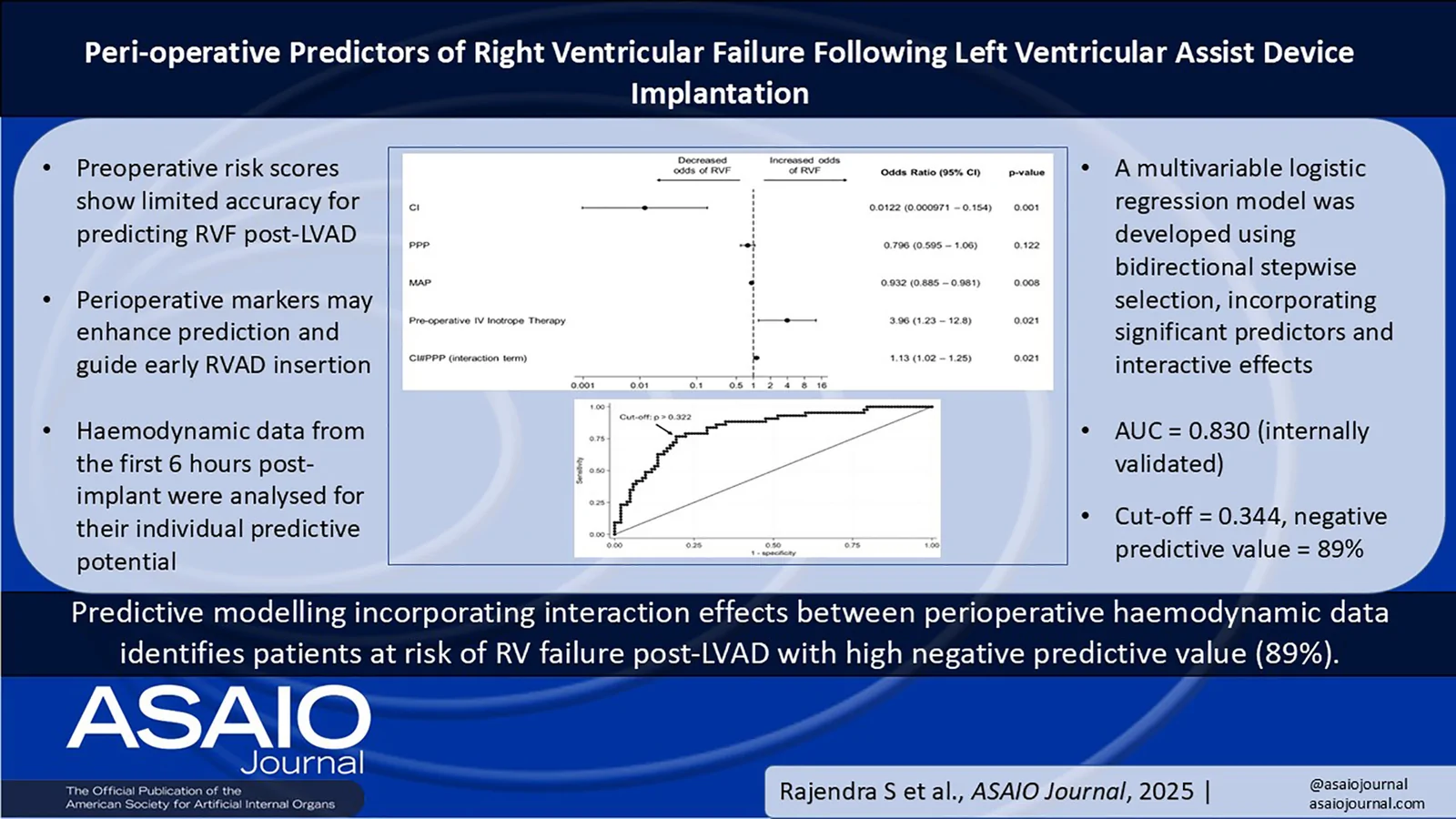

Left ventricular assist device (LVAD) implantation is increasingly being used to treat patients with advanced heart failure, as a bridge-to-transplantation or destination therapy.^1,2^ However, right ventricular failure (RVF) remains an important complication affecting ~15–45% of patients following implantation of a durable LVAD.^3–5^ Right ventricular failure leads to increased mortality, prolonged intensive care unit stays, and reduced success of bridging to transplantation, with ~5% of patients requiring a right ventricular assist device (RVAD).^3–5^

Research to date has primarily focused on preoperative predictors of RVF to better guide the selection of patients for biventricular assist therapy, which yields better outcomes compared to durable LVAD that later requires temporary RVAD support.^6^ However, preoperative echocardiographic, hemodynamic, and clinical predictors have shown limited discriminatory power in subsequent validation studies.^7,8^ The early postimplantation period may represent a crucial window for detecting hemodynamic values that might predict RVF post-LVAD because postoperative RVAD is associated with several risks, including re-sternotomy, infection, transfusion-related sensitization, and end-organ dysfunction.^7,8^ Therefore, incorporating postimplantation hemodynamic predictors into current risk models may better enable timely RVAD intervention, thus improving overall outcomes. Earlier studies have already identified some postimplantation hemodynamic parameters with potential predictive value, including cardiac index (CI), central venous pressure (CVP), pulmonary arterial pulsatility index (PAPi), mean arterial pressure (MAP), and right ventricular stroke work index (RVSWi).^9–12^ However, these were limited by small sample sizes, and the authors concluded that these are hypothesis-generating, highlighting a need for larger confirmatory studies.^10–12^

Furthermore, the complex multifactorial nature of RVF may mean that traditional modeling approaches, which assess individual predictors and their additive effects, may be insufficient for accurately capturing the complex underlying predictive relationships.^10,13–16^ Interaction effects occur when the impact of one predictor on an outcome depends on another predictor, meaning the combined effect of two variables may differ from the sum of their individual effects.^16^ Incorporating such interactions into predictive models could enhance predictive accuracy, as demonstrated in other fields.^17–20^ Translating this into the prediction of RVF post-LVAD may show promising results.

This study aims to: 1) explore key hemodynamic markers in the early post-implant phase as predictors of RVF following LVAD; 2) develop a risk scoring model incorporating both individual and interaction effects.

## Materials and Methods

### Study Design and Patient Selection

All patients who underwent HeartWare or HeartMate LVAD implantations at a large-volume tertiary care center between March 2013 and March 2023 were evaluated. Exclusion criteria included: 1) pump exchanges; 2) total artificial heart (TAH); 3) LVAD implantations that were not HeartWare or HeartMate; 4) preplanned or intraoperative need for RVAD support. Of 211 eligible patients, 1 was excluded due to inadequate data, yielding a final cohort of 210 patients.

### Outcomes

The primary outcome was early post-implant RVF, defined as one of the following within 14 days of LVAD implantation: 1) postoperative RVAD support; 2) inability to wean off inotropic support; 3) death while still on inotropic support. Inotropic support was defined as the administration of milrinone, adrenaline, isoprenaline, dobutamine, or dopamine at any dosage.

The 210 patients in the study cohort were divided into two groups: early post-implant RVF (n = 73) and non-early post-implant RVF (n = 137). For simplicity, these will be referred to as RVF and non-RVF groups throughout.

### Data Collection

Core data were prospectively collected by clinicians and recorded in Clinical Data Warehouse, a centrally managed database. These data were analyzed retrospectively. Additionally, preoperative blood test results, preoperative echocardiographic data, and preimplantation and postimplantation hemodynamic data were retrospectively collected from clinical systems and analyzed.

Preoperative and postoperative hemodynamic data were averaged over the last 6 hours before LVAD flow commenced and the first 6 hours after LVAD flow commenced, respectively.

### Statistical Analysis

All statistical analyses were performed using STATA version BE/18.0. Missing data were managed appropriately by statistical software using listwise deletion. *p* Value of less than 0.05 was considered statistically significant for final multivariate analyses, although a less stringent threshold of *p* value of less than 0.1 was applied during the earlier univariate analyses to avoid prematurely excluding potentially significant variables.

Data for the RVF and non-RVF groups were compared. Non-parametric data are presented as counts and percentages, whereas continuous variables are presented as mean ± standard deviation if normally distributed, or median with interquartile range otherwise. The Shapiro–Wilk test was used to assess normality, complemented by *z*-scores for skewness and kurtosis and visual inspection of QQ plots.

Mortality, postoperative ITU stay, and postoperative total hospital stay were compared between the two groups. Survival was assessed using Kaplan–Meier curves and the log-rank test. Length of postoperative ITU stay and hospital stay were analyzed using Poisson regression because they were count data and non-normally distributed.

### Initial Exploratory Testing

Baseline characteristics were assessed using univariate logistic regression to identify significant (*p* < 0.05) baseline confounders.

Six key post-implant parameters (CI, CVP, PAPi, MAP, RVSWi, and pulmonary pulse pressure [PPP]) were then analyzed using univariate logistic regression. Due to the study’s exploratory nature, each parameter and its potential two-way interactions were analyzed to pinpoint potential predictors of RVF post-LVAD.

### Model Selection Process and Calibration

Postimplantation hemodynamic parameters (both individual and interaction effects) with *p* values of less than 0.1 on univariate analysis were included in the multivariate model-building process, alongside the significant baseline confounders.

A stepwise bidirectional (forward and backward) selection strategy was used. Variables were iteratively added one at a time, starting with the lowest *p* value. The model was reevaluated after each addition, and variables with *p* value of greater than 0.1 were removed, continuing until no further changes were needed. A final refinement step to ensure all incorporated variables had *p* value of less than 0.05 was unnecessary, as they naturally met this criterion.

Goodness-of-fit for each model iteration was assessed using multiple metrics:

1. Akaike information criterion (AIC) and Bayesian information criterion (BIC): Lower values indicate a more parsimonious model without sacrificing explanatory power. Bayesian information criterion was prioritized to reduce the risk of overfitting, because it has a stronger penalty for complexity.
2. Log-likelihood function and pseudo *R*^2^: Higher values suggest greater explanation of the variance in the outcome. Less emphasis was placed on these metrics as they tend to favor models with a greater number of variables, thus causing overfitting.
3. Area under curve (AUC) from receiver operating characteristic (ROC) curve: Higher values reflect greater discriminatory power.
4. Hosmer–Lemeshow test: A nonsignificant result (*p* > 0.05) suggests a well-calibrated model.

Models with the best goodness-of-fit metrics were identified, with preference for models with fewer variables and interactions to reduce complexity and overfitting. The selected model then underwent *post hoc* analysis.

Preimplantation echocardiographic, hemodynamic, and blood test values were first analyzed using univariate logistic regression to identify potentially significant pre-implant variables. These were incorporated into the selected multivariate model one at a time. A decrease in AIC or BIC of greater than or equal to 2 was predetermined as a significantly improved model fit, which warrants inclusion of that variable in the finalized model.

The final model included five variables, thus adhering to the greater than 10 events per variable rule because our dataset allows for up to seven variables.^21^ One nonsignificant variable was retained in the final model because it was part of a significant interaction effect pair, to allow for a reliable interpretation of the full model.^22^

Receiver operating characteristic analysis of the final model assessed its discriminative ability by measuring the AUC value. The Hosmer–Lemeshow test confirmed model calibration. Pairwise correlation coefficients were used to assess for multicollinearity, with values greater than 0.8 indicating significant correlation.

### Multi-Seed “*k*-Fold Cross-Validation”

Finally, multi-seed five-fold cross-validation was performed to assess model robustness and sensitivity to data partitioning. The dataset was partitioned into five equal folds. In each validation round, one fold served as the validation set and the remaining four as the training set. This process was repeated five times, with each fold serving as the validation set once. To reduce variability from data shuffling, five randomly generated seeds (41,565, 17,905, 56,837, 8,862, and 70,106) were applied, yielding 25 cross-validation rounds.

## Results

Between March 2013 and March 2023, 210 patients underwent LVAD implantations at a large-volume tertiary care center. Among these, 73 (34.8%) patients developed early post-implant RVF following LVAD. Of these 73 patients, 11 (15.1%) met the RVF definition through need for postoperative RVAD support, 46 (63.0%) through prolonged inotrope dependence (> 14 days), and the remaining 16 (21.9%) through death within the first 14 days while still on inotropes. The average age of the cohort was 51.0 (39.8–58.0) years (median (interquartile range [IQR]), with males comprising 76.2% of the group. A total of 93.8% of patients were on a bridge-to-transplant strategy. Twenty patients (9.52%) received a HeartMate III LVAD, whereas the remaining 190 patients (90.5%) received a HeartWare LVAD.

### Postoperative Outcomes for Right Ventricular Failure *Versus* Non-Right Ventricular Failure Groups

Postoperative mortality was significantly higher in the RVF group (log-rank test chi² = 39.3, *p* < 0.0001). Survival was significantly lower in RVF *versus* non-RVF patients at 30 days (65.8% *vs.* 97.8%) and 1 year (46.6% *vs.* 84.7%), respectively (Figure 1). Additionally, postoperative ITU (Intensive Therapy Unit) stays were significantly longer in RVF patients (median [IQR]: 22.0 [13.0–36.0] *vs.* 8.00 [6.00–15.5] days; coefficient: 0.689, 95% confidence interval [CI]: 0.626–0.752, *p* < 0.001). However, there was no significant difference in total postoperative hospital stays between the RVF and non-RVF groups (median [IQR]: 49.0 [13.0–87.5] *vs.* 42.0 [28.0–59.5] days), coefficient: 0.0263; 95% CI: −0.00954 to 0.0621; *p* = 0.150) (Table S1, Supplemental Digital Content, https://links.lww.com/ASAIO/B768).

**Figure 1.**
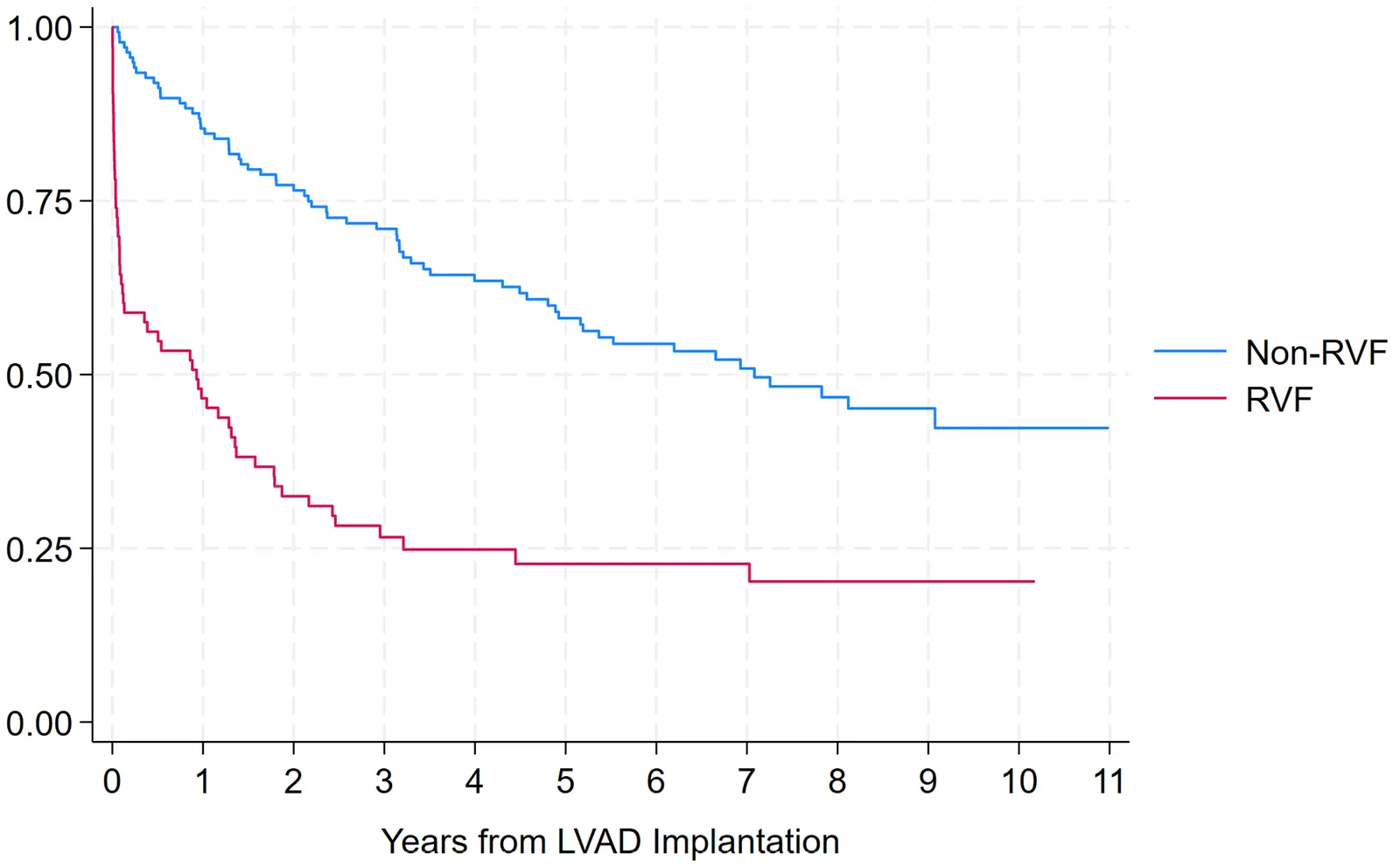
Kaplan–Meier survival curves for RVF group *vs*. non-RVF group. This figure displays Kaplan–Meier survival curves comparing the survival rates of patients in the RVF group (n = 73) and the non-RVF group (n = 137). The log-rank test shows a significant difference between the two groups (chi-squared statistic = 39.3, *p* < 0.0001). The analysis censored events at the date of mortality or the final follow-up date of March 1, 2024. Thirty day survival was significantly lower in the RVF group (65.8%) compared to the non-RVF group (97.8%). Similarly, 1 year survival was also significantly lower in the RVF group (46.6%) compared to the non-RVF group (84.7%). RVF, right ventricular failure.

### Baseline Patient Characteristics—Identifying Baseline Confounders

Baseline demographics, comorbidities, and clinical variables were compared between the RVF and non-RVF groups (Table 1). Older age (odds ratio [OR]: 1.03; 95% CI: 1.01–1.06; *p* = 0.013) and preoperative IV inotrope therapy (OR: 3.05; 95% CI: 1.33–7.00; *p* = 0.008) were significantly associated with RVF (Table 1). These baseline confounders were put forth for the multivariate model-building process.

**Table 1.** Baseline Characteristics in RVF Group *vs*. Non-RVF Group and Univariate Logistic Regression to Predict RVF Following LVAD

Variable	RVF (n = 73)	Non-RVF (n = 137)	Univariate Logistic Regression		
			Odds Ratio	95% CI	*p* Value
Age (years)	**54.0 (44.5–60.0**)	**50.0 (37.0–56.0**)	**1.03**	**1.01–1.06**	**0.013**
Gender (n, %)			1.35	0.699–2.59	0.374
Male	53 (72.6)	107 (78.1)			
Female	20 (27.4)	30 (21.9)			
Ethnicity (n, %)					
White	49 (67.1)	93 (67.9)	–	–	–
Black/Black-British	7 (9.59)	14 (10.2)	2.35	0.928–5.96	0.071
Asian/Asian-British	6 (8.22)	15 (10.9)	0.551	0.191–1.59	0.592
Other	1 (1.37)	1 (0.730)	N/A	N/A	N/A
Height (m)	1.71 ± 0.0855	1.74 (1.67–1.80)	0.979	0.950–1.01	0.177
Weight (kg)	79.0 (70.0–87.2)	82.0 (69.0–94.0)	0.993	0.977–1.01	0.371
BMI (kg/m^2^)	26.9 (23.7–29.4)	26.8 (23.7–30.7)	0.990	0.948–1.03	0.634
BSA (m^2^)	1.90 (1.80–2.10)	2.00 (1.80–2.10)	0.477	0.149–1.53	0.213
Blood group (n, %)					
Group O	32 (43.8)	51 (37.2)	–	–	–
Group A	21 (28.8)	48 (35.0)	0.697	0.354–1.37	0.297
Group B	10 (13.7)	24 (17.5)	0.319	0.0356–2.85	0.307
Group AB	1 (1.37)	5 (3.65)	0.664	0.281–1.57	0.351
INTERMACS status (n, %)			0.895	0.634–1.26	0.526
Profile 1	19 (26.0)	36 (26.3)			
Profile 2	32 (43.8)	60 (43.8)			
Profile 3	14 (19.2)	30 (21.9)			
Profile 4–7	2 (2.74)	6 (4.38)			
Ischemic heart disease (n, %)	23 (31.5)	40 (29.2)	1.20	0.643–2.25	0.564
Previous acute MI (n, %)	23 (31.5)	39 (28.5)	1.27	0.671–2.39	0.467
Diabetes (n, %)	12 (16.4)	21 (15.3)	1.14	0.524–2.49	0.737
COPD (n, %)	0 (0.00)	3 (2.19)	N/A		
Ascites (n, %)	0 (0.00)	1 (0.730)	N/A		
Symptomatic peripheral vascular disease (n, %)	2 (2.74)	0 (0.00)	0.889	0.490–1.61	0.698
Previous neurovascular event (n, %)	6 (8.22)	6 (4.38)	1.90	0.580–6.20	0.290
ICD at implant (n, %)	43 (58.9)	68 (49.6)	1.77	0.936–3.35	0.079
Previous IABP (n, %)	16 (21.9)	19 (13.9)	1.85	0.872–3.90	0.109
Preoperative IV inotrope therapy (n, %)	**57 (78.1**)	**91 (66.4**)	**3.05**	**1.33–7.00**	**0.008**
Concomitant surgery (n, %)	4 (5.48)	8 (5.84)	0.984	0.285–3.39	0.980
Previous sternotomy (n, %)	10 (13.7)	17 (12.4)	1.19	0.506–2.79	0.692

This table presents the baseline characteristics for patients in the RVF and non-RVF groups. Non-parametric data are presented as counts and their corresponding percentages. Continuous data are presented as mean ± standard deviation if normally distributed, or median with IQR for non-normally distributed data. Results of univariate logistic regression to identify variables that are significantly associated (*p* < 0.05) with RVF following LVAD are presented. Their odds ratios, 95% confidence intervals, and *p* values are provided. Statistically significant results are bolded. N/A refers to variables where perfect prediction prevented these values from being obtained.

BMI, body mass index; BSA, body surface area; COPD, chronic obstructive pulmonary disease; IABP, intra-aortic balloon pump; ICD, implantable cardioverter device; MI, myocardial infarction.

### Post-Implant Hemodynamic Predictors—Univariate Analysis

Postimplantation hemodynamic parameters averaged over the first 6 hours of implantation for the two groups were assessed using univariate logistic regression (Table 2). Patients with lower post-implant CI (OR: 0.294; CI: 0.157–0.553; *p* < 0.001) and lower MAP (OR: 0.934; CI: 0.899–0.970; *p* < 0.001) had significantly greater odds of RVF. Central venous pressure was a near-significant (*p* < 0.1) predictor of RVF, whereas PPP, PAPi, and RVSWi showed no significant association with RVF (Table 2).

**Table 2.** Post-Implant Hemodynamic Results in RVF Group *vs*. Non-RVF Group and Univariate Logistic Regression to Predict RVF Following LVAD

Variable	RVF (n = 73)	Non-RVF (n = 137)	Univariate Logistic Regression		
			Odds Ratio	95% CI	*p* Value
CI (L/min/m^2^)	**2.40 (2.00–2.80**)	**2.80 (2.50–3.45**)	**0.294**	**0.157–0.553**	**< 0.001**
CVP (mm Hg)	14.1 ± 3.52	13.0 (11.0–15.0)	1.08	0.998–1.18	0.056
PPP (mm Hg)	17.2 (12.0–23.2)	18.6 ± 6.72	0.988	0.948–1.03	0.568
PAPi	1.21 (0.795–1.72)	1.41 (0.906–1.88)	0.741	0.463–1.19	0.212
RVSWi (g·m/m^2^/beat)	0.440 (0.265–0.690)	0.549 (0.362–0.726)	0.652	0.265–1.60	0.351
MAP (mm Hg)	**65.7 ± 7.80**	**70.2 ± 8.57**	**0.934**	**0.899–0.970**	**< 0.001**

This table presents the post-implant hemodynamic parameters (averaged over the first 6 hours post-LVAD implantation) for patients in the RVF and non-RVF groups. Data are presented as mean ± standard deviation if normally distributed, or median with IQR for non-normally distributed data. Results of univariate logistic regression to identify significant predictors (*p* < 0.05) and near-significant predictors (*p* < 0.1) of RVF following LVAD are presented. Their odds ratios, 95% confidence intervals, and *p* values are provided. Statistically significant results are bolded.

CI, cardiac index; CVP, central venous pressure; MAP, mean arterial pressure; PAPi, pulmonary artery pulsatility index; PPP, pulmonary pulse pressure; RVSWi, right ventricular stroke work index.

Univariate logistic regression analysis of two-way interaction terms revealed a significant interaction between CI and PPP (OR: 1.13; CI: 1.03–1.23; *p* = 0.007), indicating the relationship between CI and RVF risk depends on PPP levels (Figure 2A). At low PPP levels, a higher CI substantially reduces RVF risk (*eg*, with PPP at 9 mm Hg, the RVF probability is 0.591 at CI = 2, dropping sharply to 0.00240 at CI = 4). Conversely, as PPP increases, the protective impact of higher CI on RVF risk diminishes (*eg*, with PPP at 33 mm Hg, the RVF probability is 0.566 at CI = 2 and 0.412 at CI = 4). Other interactions were near-significant (*p* < 0.1) predictors, including CI and MAP (Figure 2B), RVSWi and MAP (Figure 2C), MAP and PPP (Figure 2D), and CI and PAPi (Figure 2E). All other interaction effects tested were nonsignificant (Table 3).

**Table 3.** Univariate Logistic Regression of Post-Implant Hemodynamic Interaction Effects to Predict RVF Following LVAD

Interaction	Univariate Logistic Regression		
	Odds Ratio	95% CI	*p* Value
CI and PPP	**1.13**	**1.03–1.23**	**0.007**
CI and PAPi	2.50	0.895–6.95	0.080
CI and CVP	1.10	0.934–1.30	0.247
CI and MAP	0.921	0.847–1.00	0.051
CVP and PPP	1.01	0.994–1.02	0.281
CVP and MAP	0.998	0.988–1.01	0.744
PPP and MAP	0.994	0.987–1.00	0.087
PAPi and MAP	0.979	0.913–1.05	0.555
RVSWi and MAP	0.866	0.744–1.01	0.061

This table presents the results of univariate logistic regression assessing interaction effects between pairs of post-implant hemodynamic parameters to predict RVF following LVAD. Interaction effects explore how the impact of one predictor on the outcome depends on the level of another predictor. Odds ratios, 95% confidence intervals, and *p* values for each interaction effect tested are provided. Statistically significant results are bolded.

CI, cardiac index; CVP, central venous pressure; MAP, mean arterial pressure; PAPi, pulmonary artery pulsatility index; PPP, pulmonary pulse pressure; RVSWi, right ventricular stroke work index.

**Figure 2.**
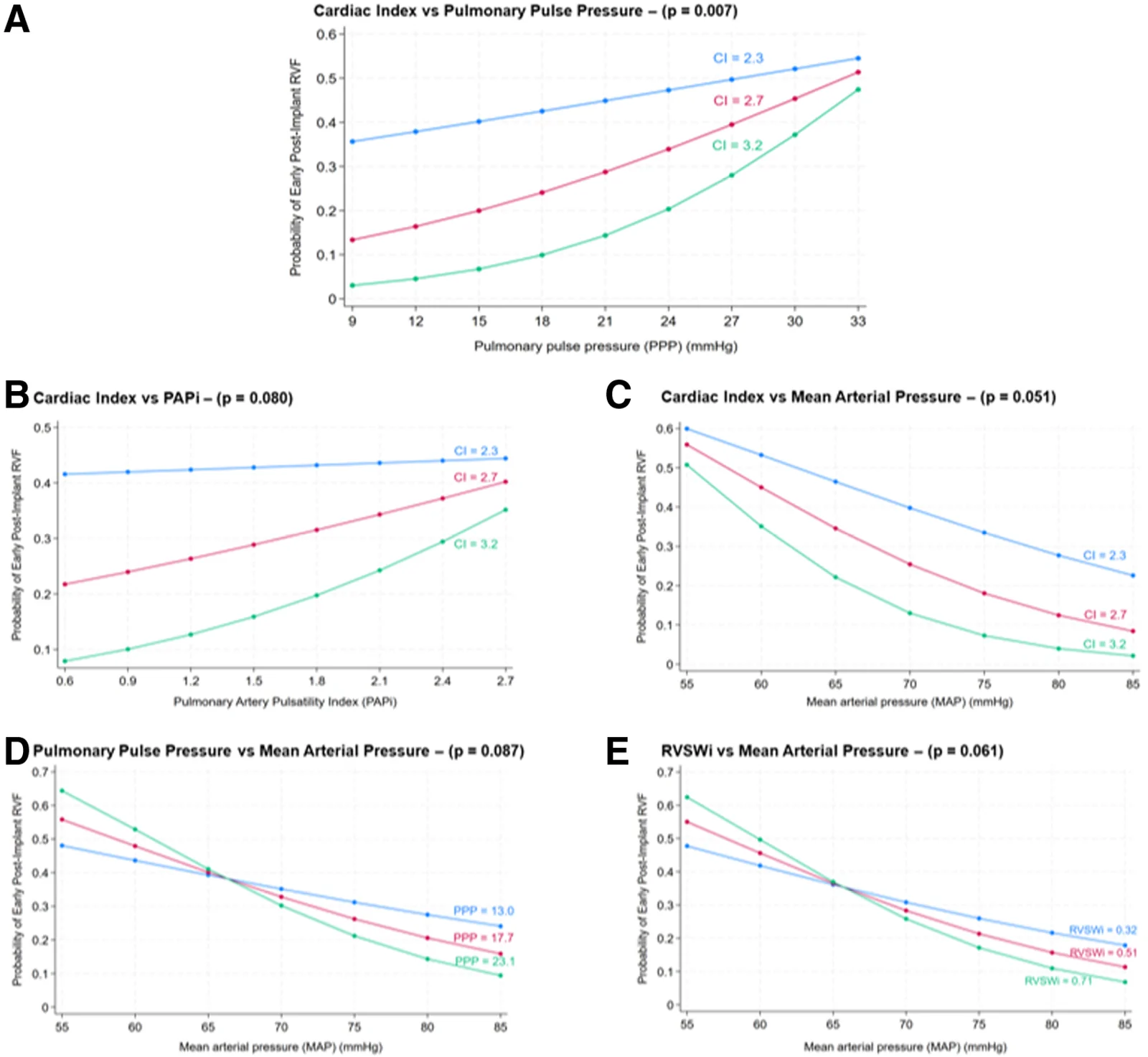
Interaction plots of significant and near-significant interaction effects between post-implant hemodynamic parameters for predicting RVF following LVAD. This figure presents interaction plots, which illustrate the impact of one variable on the ability of another variable to predict the outcome. Significant (*p* < 0.05) and near-significant (*p* < 0.1) interaction effects between post-implant hemodynamic parameters for predicting RVF following LVAD are shown: (**A**) varying PPP at fixed CI levels; (**B**) varying PAPi at fixed CI levels; (**C**) varying MAP at fixed CI levels; (**D**) varying MAP at fixed PPP levels; (**E**) varying MAP at fixed RVSWi levels. The *x*-axis presents the middle 90% of observed data (to reduce the impact of outliers). Each line corresponds to the lower quartile (Q1), median (Q2), and upper quartile (Q3) of the interacting variable. The *p* value in the title of each graph corresponds to the *p* value of that interaction effect when predicting RVF via univariate logistic regression. CI, cardiac index; LVAD, left ventricular assist device; MAP, mean arterial pressure; PPP, pulmonary pulse pressure; RVF, right ventricular failure; RVSWi, right ventricular stroke work index.

### Multivariate Analysis—Model Selection

All significant and near-significant (*p* < 0.1) post-implant hemodynamic predictors identified from univariate analyses, along with baseline confounders, were entered into a stepwise bidirectional multivariate logistic regression. Ten models (model 1 to model 10) were developed with varying combinations of variables and interaction effects. Performance metrics for each model were calculated (Table 4). Models 4, 9, and 10 showed better goodness-of-fit compared to the others. Model 4 was selected for its simplicity and lower risk of overfitting, containing five variables with one interaction term. It achieved an AUC of 0.830, close to model 9’s AUC of 0.853 (seven variables with two interaction terms) and model 10’s AUC of 0.848 (six variables with two interaction terms) (Table 4). Model 4 is detailed as a forest plot in Figure 3.

**Table 4.** Summary of Components and Performance Metrics for Multivariate Logistic Regression Models to Predict RVF Post-LVAD

	Model 1	Model 2	Model 3	Model 4	Model 5	Model 6	Model 7	Model 8	Model 9	Model 10
CI	**X**	**X**	**X**	**X**	**X**	**X**	**X**	**X**	**X**	**X**
CVP							**X**			
PPP			**X**	**X**	**X**	**X**	**X**	**X**	**X**	**X**
PAPi								**X**		
RVSWi									**X**	
MAP		**X**	**X**	**X**	**X**	**X**	**X**	**X**	**X**	**X**
Age					**X**					
Preoperative IV inotrope therapy				**X**	**X**	**X**	**X**	**X**	**X**	**X**
CI and PPP (interaction)			**X**	**X**	**X**	**X**	**X**	**X**	**X**	**X**
CI and PAPi (interaction)								**X**		
RVSWi and MAP (interaction)									**X**	
PPP and MAP interaction										**X**
CI and MAP interaction						**X**	**X**	**X**		
AIC	187.2	179.5	164.7	144.6	145.0	142.6	143.4	143.2	139.4	141.2
BIC	193.4	188.8	180.0	162.5	165.9	163.5	167.3	170.0	163.1	162.1
Log-likelihood	−91.6	−86.8	−77.3	−66.3	−65.5	−64.3	−63.7	−62.6	−61.7	−63.6
Pseudo *R*^2^	0.0990	0.147	0.228	0.251	0.260	0.274	0.280	0.290	0.295	0.282
AUC	0.701	0.742	0.809	0.830	0.834	0.840	0.847	0.852	0.853	0.848
Hosmer Lemeshow Chi-square, *p* value	3.38, 0.908	3.75, 0.879	2.19, 0.975	5.35, 0.720	8.92, 0.350	4.61, 0.798	7.61, 0.473	3.02, 0.933	5.09, 0.748	12.5, 0.131

This table summarizes the variables included in each of the 10 models (model 1 to model 10) that were developed through the multivariate stepwise bidirectional logistic regression process to predict RVF post-LVAD. Their respective performance metrics have also been provided. The variables included in this model-building process include significant (*p* < 0.05) and near-significant (*p* < 0.1) post-implant hemodynamic parameters from univariate analysis, as well as age and preoperative IV inotrope therapy, which were baseline confounders. The stepwise logistic regression process continued until no further variables could be added or removed. The following performance metrics for all 10 models were compared: 1) AIC; 2) BIC; 3) Log-likelihood; 4) Pseudo *R*^2^; 5) AUC; 6) The Hosmer–Lemeshow Chi-square, *p* value.

AIC, akaike information criterion; AUC, area under the curve; BIC, Bayesian information criterion; CI, cardia index; CVP, central venous pressure; PPP, pulmonary pulse pressure; PAPi, pulmonary artery pulsatility index; RSVWi, right ventricular stroke work index; MAP, mean arterial pressure.

**Figure 3.**
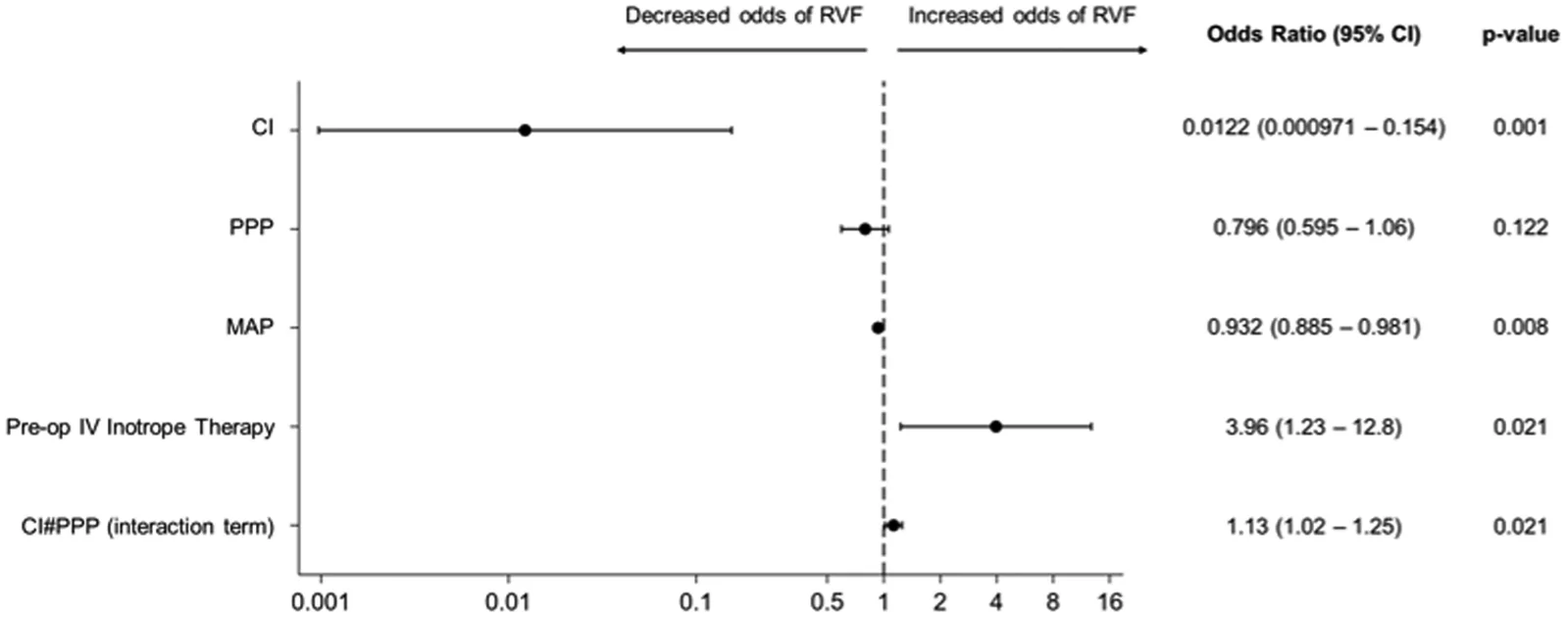
Multivariate logistic regression results for the candidate model predicting RVF following LVAD. This figure displays the multivariate logistic regression results of the selected candidate model (model 4) as a forest plot. Odds ratios, 95% confidence intervals, and *p* values for each interaction effect tested are provided. Each circle on the plot represents the odds ratio for that predictor, with horizontal lines depicting the confidence intervals. The *x*-axis of the forest plot is on a logarithmic scale. CI, cardiac index; LVAD, left ventricular assist device; MAP, mean arterial pressure; PPP, pulmonary pulse pressure.

### Post Hoc Testing

Univariate analysis revealed the following preoperative variables significantly increased the risk of RVF post-LVAD, greater than or equal to moderate tricuspid regurgitation, lower lymphocyte count, lower heart rate, lower SpO_2,_ and lower MAP (Table S2, Supplemental Digital Content, https://links.lww.com/ASAIO/B768). *Post hoc* testing revealed that adding these five preoperative variables individually to the final model did not significantly improve model fit (Table S3, Supplemental Digital Content, https://links.lww.com/ASAIO/B768). Furthermore, removing the CI and PPP interaction term significantly worsened model fit (as demonstrated by the increase in AIC by 4). Thus, keeping the interaction term in the predictive model was justified despite the added complexity.

The Hosmer–Lemeshow test yielded a nonsignificant result (chi-squared statistic = 5.35; *p* = 0.720), indicating the model is well-calibrated, with no significant deviation between observed outcomes and predictions made by the model across different subgroups. Moreover, pairwise correlation coefficients among variables in the multivariate model revealed no significant correlations, confirming the absence of multicollinearity (Table S4, Supplemental Digital Content, https://links.lww.com/ASAIO/B768).

### Receiver Operating Characteristic Curve Assessment and Risk Cutoff

Our proposed risk score, based on the selected logistic regression model, is calculated as follows:


$$\begin{array}{ll} \; & Logit\left(\pi \right)=12.6\;\left(4.40\times CI\right)\;\left(0.0706\times MAP\right)\;\left(0.229\times PPP\right)\\ \; & +\left(0.121\times CI\times PPP\right)+(1.38\times pre-implant\;IV\;inotrope\;therapy) \end{array}$$

A patient’s predicted probability of RVF post-LVAD is then calculated from the risk score as:


$$Predicted\;probability=\frac{e^{Logit}}{1+e^{Logit}}$$

Our model achieves an AUC of 0.830 (95% CI: 0.757–0.902), demonstrating strong potential to distinguish between RVF and non-RVF patients (Figure 4). A probability threshold of greater than 0.344, identified via Youden’s index, was established as a cutoff for classifying patients as high-risk for RVF post-LVAD (sensitivity: 76.7%, specificity: 80.6%, positive predictive value [PPV]: 62.3%, negative predictive value [NPV]: 89.2%) (Figure 4, Table S5, Supplemental Digital Content https://links.lww.com/ASAIO/B768).

**Figure 4.**
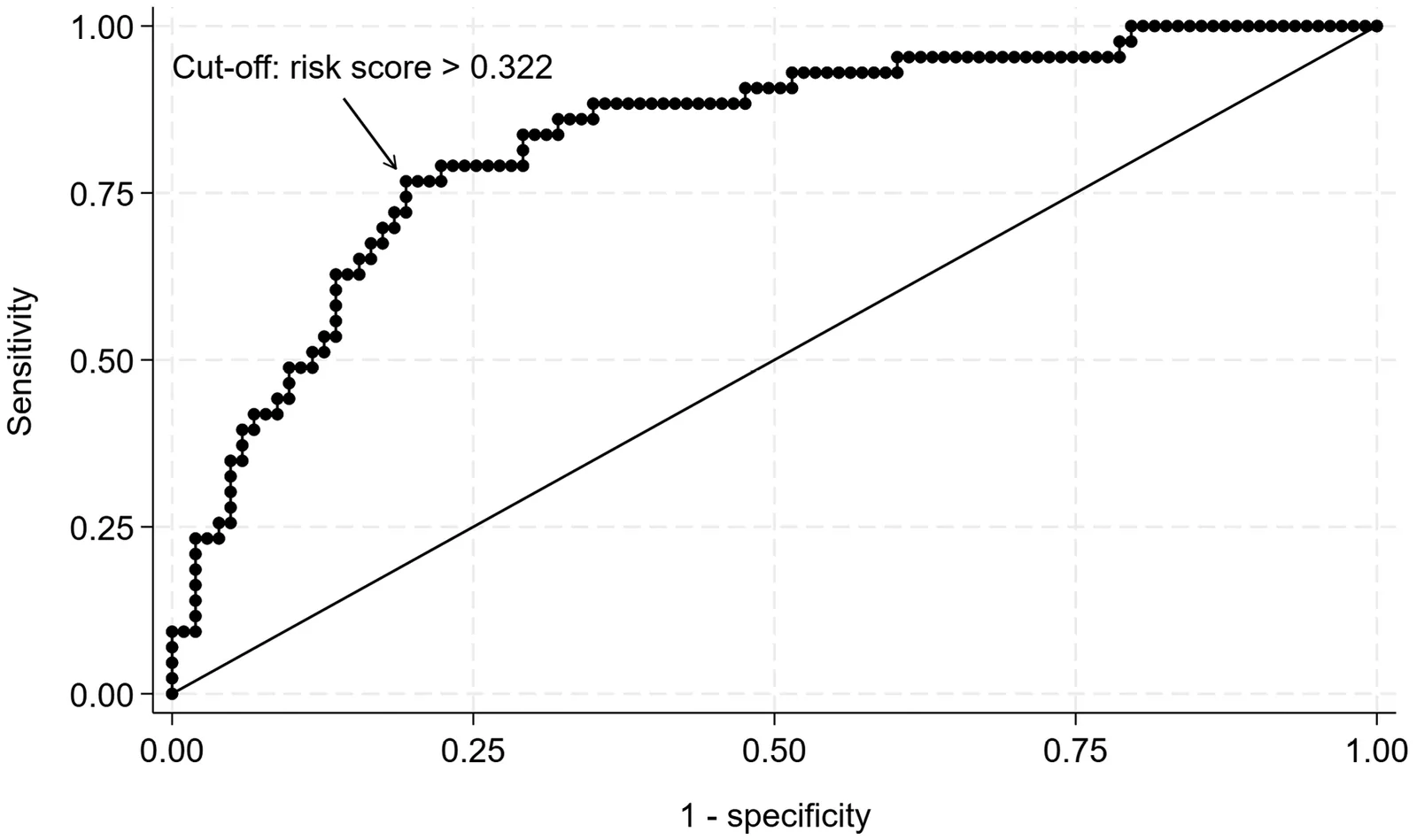
ROC curve for the proposed predictive model of RVF following LVAD. This figure displays the ROC curve demonstrating the discriminative ability of the predictive model developed to identify patients at risk of RVF following LVAD. The AUC is 0.830, which suggests a strong predictive performance. The curve shows the trade-off between sensitivity (true positive rate) and specificity (false positive rate) at various thresholds. Youden’s index was used to choose the risk probability cutoff in this study (*p* > 0.322), which is marked by an arrow. AUC, area under the curve; LVAD, left ventricular assist device; ROC, receiver operating characteristic; RVF, right ventricular failure.

### Cross-Validation

Multi-seed five-fold cross-validation confirmed model robustness and stability. Across 25 cross-validation rounds, the mean cross-validated AUC was 0.806, closely matching the initially obtained AUC of 0.830, indicating stability and reliability of the model across various data splits. The lack of significant differences in sensitivity, specificity, PPV, and NPV between the overall model and the cross-validation averages further suggests that the model is not unduly influenced by specific data segments (Table S5, Supplemental Digital Content, https://links.lww.com/ASAIO/B768).

## Discussion

This study enhances our current understanding of post-implant hemodynamic predictors of RVF following LVAD. In a larger cohort of 210 patients, we confirm findings from smaller studies (n = 33–110) and introduce new insights into the use of statistical interaction effects between hemodynamic variables for predicting RVF risk.^9–12^

### Confirming and Expanding on Previous Findings

Consistent with prior research, RVF was associated with significantly lower survival and prolonged ITU stays.^3–5^ Postimplantation MAP and CI emerged as significant predictors of RVF post-LVAD, supporting findings by Cordtz *et al.*,^10^ who identified both variables as having significant predictive value at ICU admission. Notably, the Cordtz *et al*.^10^ study found that only MAP maintained its predictive value at 12 hours postoperatively, whereas we averaged the values over the first 6 hours post-LVAD flow initiation and found both CI and MAP to be significant.^10^ Although earlier studies found RVSWi, PAPi, and CVP to be significant predictors, our study did not.^9,11,12^ This may reflect differences between single-timepoint measurements versus averaged values. Nonetheless, Wei et al found that PAPi averaged over the first 48 hours postoperatively was significantly predictive, emphasizing that the measurement timeframe influences accuracy.^9^ Our findings underline the necessity for future research to not only identify predictors but also establish the most accurate temporal window for measurements to enhance predictive accuracy.

### Novel Insights

Exploring interaction effects yielded novel insights. The interaction between CI and PPP was significant for predicting RVF. Individually, higher CI reduced RVF risk, whereas PPP had no independent effect, but concurrent increases in both CI and PPP led to increased RVF risk, suggesting that PPP modifies CI’s impact on RVF risk. At lower PPP, higher CI was protective against RVF, but this benefit diminished as PPP increased. Overall, we demonstrate that incorporating interaction effects into predictive modeling significantly improved accuracy, supporting further work in this area.

The chosen risk score cutoff (*p* > 0.344) balances high sensitivity and specificity. The high NPV (89.2%) suggests the proposed model can reliably exclude RVF post-LVAD, although the modest PPV (62.3%) raises the risk of false positives.

### Limitations and Future Directions

This study has several limitations. Although internal cross-validation supports the robustness of our model, external validation with multicenter cohorts is needed to confirm reproducibility. Future studies should also compare this model with established pre-implant predictors to assess incremental accuracy, thus justifying its clinical utility.

No adjustments were made for multiple comparisons, increasing the risk of type I errors. Our cohort was predominantly HeartWare LVADs, with a few HeartMate 3 devices. Although both are centrifugal continuous-flow LVADs, differences in pump design may confound results and limit generalisability to centers using other LVAD devices. Future studies should include a broader range of LVAD types. In addition, postoperative hemodynamic parameters were averaged over the first six hours post-LVAD implantation. Although this reduced variability, it may have smoothed short-term fluctuations that may potentially be prognostically significant. Time-series analyses using higher resolution data may better characterize early signs of impending RVF.

Finally, a recurring challenge in this field is the inconsistent definitions of RVF post-LVAD across studies, which complicates inter-study comparisons.^8^ The definition of RVF used in this study includes patients who died early while still on inotropic support. Although this aligns with previously published studies, it may also capture patients with multi-organ or circulatory dysfunction rather than RV dysfunction specifically. Furthermore, the clinical decision to use inotropes postoperatively is directly influenced by hemodynamic parameters, and vice versa. Using these parameters as predictors may introduce an inherent bias, although this is a limitation in all recently published studies in this area.^9–12^ Ideally, prospective studies with standardized inotrope protocols are needed. Although the recent INTERMACS 2021 criteria update provides a more precise definition, no studies have yet explored this refined definition.^23^ Without a universal and reliable definition across studies, developing robust predictive models will likely remain challenging.

## Conclusions

This study demonstrates that CI and MAP in the peri-implantation period are significant predictors of RVF following LVAD, confirming earlier findings. We provide new insights into the significant interaction between CI and PPP for predicting RVF and propose a prediction model showing significant discriminatory power. Although internal cross-validation suggests the model is robust, external validation is crucial to determine reproducibility. Predictive models that accurately reflect the complex dynamics of RVF post-LVAD will enable more timely interventions and targeted management.

## Supplementary Material

## References

[1] Goldstein DJ Meyns B Xie R: Third annual report from the ISHLT mechanically assisted circulatory support registry: A comparison of centrifugal and axial continuous-flow left ventricular assist devices. J Heart Lung Transplant 38: 352–363, 2019. 10.1016/j.healun.2019.02.004 30945637

[2] Chair SY Yu DS Ng MT: Evolvement of left ventricular assist device: The implications on heart failure management. J Geriatr Cardiol 13: 425–430, 2016. 10.11909/j.issn.1671-5411.2016.05.015 PMC4984573 27594870

[3] Frankfurter C Molinero M Vishram-Nielsen JKK: Predicting the risk of right ventricular failure in patients undergoing left ventricular assist device implantation: A systematic review. Circ Heart Fail 2020: e00, 6994. 10.1161/CIRCHEARTFAILURE.120.006994 32981331

[4] de By TMMH Schoenrath F Veen KM: The European registry for patients with mechanical circulatory support of the European Association for Cardio-Thoracic Surgery: Third report. Eur J Cardiothorac Surg 62: ezac032, 2022. 10.1093/ejcts/ezac032 35150247

[5] Reid G Mork C Gahl B: Outcome of right ventricular assist device implantation following left ventricular assist device implantation: Systematic review and meta-analysis. Perfusion 37: 773–784, 2022. 10.1177/02676591211024817 PMC9619248 34112048

[6] Ahmed MM Jacobs JP Meece LE: Timing and outcomes of concurrent and sequential biventricular assist device implantation: A Society of Thoracic Surgeons Intermacs analysis. Ann Thorac Surg 116: 383–390, 2023. 10.1016/j.athoracsur.2023.02.058 36935029

[7] Rodenas-Alesina E Brahmbhatt DH Rao V Salvatori M Billia F: Prediction, prevention, and management of right ventricular failure after left ventricular assist device implantation: A comprehensive review. Front Cardiovasc Med 9: 251, 1040. 10.3389/fcvm.2022.1040251 PMC9671519 36407460

[8] Lampert BC Teuteberg JJ: Right ventricular failure after left ventricular assist devices. J Heart Lung Transplant 34: 1123–1130, 2015. 10.1016/j.healun.2015.06.015 26267741

[9] Wei J Franke J Kee A Dukes R Leonardo V Flynn BC: Postoperative pulmonary artery pulsatility index improves prediction of right ventricular failure after left ventricular assist device implantation. J Cardiothorac Vasc Anesth 38: 214–220, 2024. 10.1053/j.jvca.2023.10.010 37973507

[10] Cordtz J Nilsson JC Hansen PB: Right ventricular failure after implantation of a continuous-flow left ventricular assist device: Early haemodynamic predictors. Eur J Cardiothorac Surg 45: 847–853, 2014. 10.1093/ejcts/ezt519 24258201

[11] Gudejko MD Gebhardt BR Zahedi F: Intraoperative hemodynamic and echocardiographic measurements associated with severe right ventricular failure after left ventricular assist device implantation. Anesth Analg 128: 25–32, 2019. 10.1213/ANE.0000000000003538 PMC7908049 29878942

[12] Gebhardt BR Abdulaziz A Kawabori M Gudejko MD Cobey FC: Intraoperative central venous pressure and diastolic pulmonary artery pressure as a marker of severe right ventricular failure after left ventricular assist device implantation. J Cardiothorac Vasc Anesth 34: 847–849, 2020. 10.1053/j.jvca.2019.08.043 31570242

[13] Frankfurter C Molinero M Vishram-Nielsen JKK: Predicting the risk of right ventricular failure in patients undergoing left ventricular assist device implantation: A systematic review. Circ Heart Fail 13: e006994, 2020. 10.1161/CIRCHEARTFAILURE.120.006994 32981331

[14] Pinsky MR: The right ventricle: Interaction with the pulmonary circulation. Crit Care 20: 266, 2016. 10.1186/s13054-016-1440-0 PMC5018168 27613549

[15] Liou JW Liou M Cheng PE. An information analysis on modeling interaction effects in logistic regression. arXiv. Ithaca: Cornell University Library; 2018. https://arxiv.org/pdf/1801.01003. Accessed April 20 2024.

[16] Shipe ME Deppen SA Farjah F Grogan EL: Developing prediction models for clinical use using logistic regression: An overview. J Thorac Dis 11: S574–S584, 2019. 10.21037/jtd.2019.01.25 PMC6465431 31032076

[17] Xu EL Qian X Yu Q Zhang H Cui S: Feature selection with interactions in logistic regression models using multivariate synergies for a GWAS application. BMC Genomics 19: 170, 2018. 10.1186/s12864-018-4552-x PMC5872388 29589561

[18] Joshi RD Dhakal CK: Predicting type 2 diabetes using logistic regression and machine learning approaches. Int J Environ Res Public Health 18: 7346, 2021. 10.3390/ijerph18147346 PMC8306487 34299797

[19] Jensen SM Hauger H Ritz C: Mediation analysis for logistic regression with interactions: Application of a surrogate marker in ophthalmology. PLoS One 13: e0192857, 2018. 10.1371/journal.pone.0192857 PMC5809055 29432493

[20] Mambo A Yang Y Mahulu E Zihua Z: Investigating the interplay of smoking, cardiovascular risk factors, and overall cardiovascular disease risk: NHANES analysis 2011–2018. BMC Cardiovasc Disord 24: 193, 2024. 10.1186/s12872-024-03838-7 PMC10993506 38575889

[21] Peduzzi P Concato J Kemper E Holford TR Feinstein AR: A simulation study of the number of events per variable in logistic regression analysis. J Clin Epidemiol 49: 1373–1379, 1996. 10.1016/s0895-4356(96)00236-3 8970487

[22] Penn State Eberly College of Science. Interactions between quantitative predictors. STAT 501. https://online.stat.psu.edu/stat501/lesson/9/9.6. Accessed April 23, 2024.

[23] Kormos RL Antonides CFJ Goldstein DJ: Updated definitions of adverse events for trials and registries of mechanical circulatory support: A consensus statement of the mechanical circulatory support academic research consortium. J Heart Lung Transplant 39: 735–750, 2020. 10.1016/j.healun.2020.03.010 32386998

