# Supplementary material for: Perioperative Predictors of Right Ventricular Failure Following Left Ventricular Assist Device Implantation
Source: ASAIO J. 2025 Dec 23;72(11):905–13. doi: 10.1097/MAT.0000000000002631 (PMC13704692; doi:10.1097/MAT.0000000000002631)
Supplement: Supplementary file 1 [file mat-72-0905-s001.pdf]

Supplemental Digital Content

Table S1 – Post-operative outcomes for RVF vs non-RVF groups

Variable	RVF (n = 73)	Non-RVF (n = 137)	Poisson Regression		
			Coefficient	95% CI	p-value
**Post-operative length of ITU stay (days)**	**22.0 (13.0–36.0)**	**8.00 (6.00–15.5)**	**0.689**	**0.626 – 0.752**	**<0.001**
Post-operative length of hospital stay (days)	49.0 (13.0–87.5)	42.0 (28.0–59.5)	0.0263	-0.00954 – 0.0621	0.150

This table presents the post-operative length of ITU stay and post-operative length of hospital stay, for the RVF and non-RVF groups. Data are presented as median with interquartile range (IQR) as they are non-normally distributed. Results of poisson regression to identify variables that are significantly associated ($p<0.05$) with RVF following LVAD are presented. Their odds ratios, 95% confidence intervals and p-values are provided. Statistically significant results are bolded.

Table S2 – Pre-operative echocardiographic, blood test and haemodynamic results for RVF vs non-RVF groups and univariate logistic regression to predict RVF following LVAD

Variable	RVF (n = 73)	Non-RVF (n = 137)	Univariate Logistic Regression		
			Odds ratio	95% CI	p-value
**Pre-operative echocardiographic results**					
Interventricular septal thickness at end-diastole (IVSd) (cm)	0.900 (0.800 – 1.10)	0.900 (0.800 – 1.06)	1.35	0.422 – 4.32	0.613
LV internal dimension in diastole (LVIDd) (cm)	6.61 ± 1.07	6.85 ± 1.08	0.812	0.618 – 1.07	0.133
LV internal dimension in systole (LVIDs) (cm)	5.95 ± 1.10	6.12 ± 1.20	0.884	0.688 – 1.14	0.334
LV posterior wall thickness at end-diastole (LVPWd) (cm)	0.900 (0.700 – 1.05)	0.820 (0.700 – 1.03)	1.25	0.766 – 2.05	0.369
Tricuspid annular plane systolic excursion (TAPSE) (cm)	1.37 ± 0.443	1.37 ± 0.436	1.03	0.537 – 1.98	0.925
Ejection fraction (EF) (%)	20.8 (14.3 – 26.8)	20.7 ± 8.24	1.01	0.977 – 1.04	0.551
LV end-diastolic volume (LV EDV) (ml)	227 ± 81.0	215 (164 – 297)	0.998	0.995 – 1.00	0.306
LV end-systolic volume (LV ESV) (ml)	168 (127 – 227)	174 (126 – 247)	0.998	0.994 – 1.00	0.170
RA size					
≥ severely dilated (n, %)	18 (24.7)	28 (20.4)	1.15	0.578 – 2.30	0.688
≥ moderately dilated (n, %)	24 (32.9)	42 (30.7)	0.980	0.522 – 1.84	0.949
LA size					
≥ severely dilated (n, %)	35 (47.9)	55 (40.1)	1.39	0.767 – 2.52	0.276
≥ moderately dilated (n, %)	39 (53.4)	69 (50.4)	1.13	0.620 – 2.06	0.689
RV size					
≥ severely dilated (n, %)	8 (11.0)	6 (4.38)	2.67	0.887 – 8.01	0.081
≥ moderately dilated (n, %)	11 (15.1)	10 (7.30)	2.24	0.900 – 5.55	0.083
LV size					
≥ severely dilated (n, %)	35 (47.9)	68 (49.6)	0.904	0.509 – 1.60	0.731
≥ moderately dilated (n, %)	43 (58.9)	87 (63.5)	0.784	0.434 – 1.42	0.420
RV function					

≥ severely impaired (n, %)	17 (23.3)	20 (14.6)	1.72	0.833 – 3.53	0.143
≥ moderately impaired (n, %)	26 (35.6)	34 (24.8)	1.61	0.868 – 3.00	0.131
LV function					
≥ severely impaired (n, %)	67 (91.8)	125 (91.2)	0.893	0.311 – 2.56	0.834
≥ moderately impaired (n, %)	70 (95.9)	130 (94.9)	0.897	0.208 – 3.87	0.885
Tricuspid regurgitation					
≥ severe regurgitation (n, %)	12 (16.4)	17 (12.4)	1.41	0.633 – 3.15	0.400
**≥ moderate regurgitation (n, %)**	**35 (47.9)**	**46 (33.6)**	**1.88**	**1.05 – 3.38**	**0.035**
Mitral regurgitation					
≥ severe regurgitation (n, %)	28 (38.4)	47 (34.3)	1.19	0.659 – 2.15	0.562
≥ moderate regurgitation (n, %)	43 (58.9)	86 (62.8)	0.845	0.845 – 0.253	0.574
Pulmonary regurgitation					
≥ severe regurgitation (n, %)	0 (0.00)	0 (0.00)	N/A	N/A	N/A
≥ moderate regurgitation (n, %)	1 (1.37)	8 (5.89)	0.228	0.0279 – 1.87	0.169
**Pre-operative blood test results**					
Haemoglobin (g/L)	133 (116 – 145)	127 ± 19.6	1.00	0.987 – 1.02	0.894
Red blood cells (RBC) ($x10^{12}$/L)	4.35 ± 0.670	4.40 ± 0.685	0.900	0.592 – 1.37	0.623
Platelets ($x10^9$/L)	192 (157 – 259)	200 (157 – 249)	1.00	1.00 – 1.00	0.910
White blood cells (WBC) ($x10^9$/L)	8.10 (6.80 – 10.9)	8.10 (6.65 – 10.6)	1.00	0.935 – 1.07	0.981
Neutrophils ($x10^9$/L)	5.80 (4.60 – 7.80)	5.60 (4.55 – 7.85)	1.01	0.942 – 1.09	0.733
**Lymphocytes ($x10^9$/L)**	**1.20 (1.00 – 1.60)**	**1.50 (1.05 – 2.00)**	**0.561**	**0.347 – 0.907**	**0.018**
Sodium (mmol/L)	135 ± 5.40	135 (132 – 138)	0.986	0.932 – 1.04	0.630
Potassium (mmol/L)	4.20 (3.85 – 4.65)	4.20 (3.88 – 4.50)	1.30	0.782 – 2.17	0.309
Urea (mmol/L)	9.30 (6.30 – 12.5)	8.20 (5.95 – 11.2)	1.05	0.983 – 1.12	0.151
Creatinine (mmol/L)	104 (75.5 – 129)	102 (73.5 – 126)	1.00	0.996 – 1.01	0.482
GFR (ml/min)	52.6 ± 17.9	58.1 ± 16.6	0.981	0.959 – 1.00	0.095
Bilirubin (µmol/min)	19.0 (13.0 – 28.5)	19.0 (12.0 – 28.0)	1.01	0.997 – 1.03	0.112
Alanine Aminotransferase (ALT) (IU/L)	24.5 (18.0 – 45.5)	30.0 (18.0 – 50.0)	1.00	0.998 – 1.00	0.944
C-reactive protein (CRP) (mg/L)	16.0 (4.50 – 79.5)	12.0 (3.00 – 45.0)	1.00	0.998 – 1.01	0.278
Brain natriuretic peptide (BNP) (ng/L)	1050 (556 – 1410)	992 (572 – 1810)	1.00	0.999 – 1.00	0.338

Troponin (ng/L)	59.9 (30.0 – 310)	128 (36.8 – 589)	1.00	1.00 – 1.00	0.675
**Pre-implant haemodynamic results**					
Pulmonary artery systolic pressure (mmHg)	33.8 ± 9.30	32.9 (27.8 – 38.2)	0.997	0.967 – 1.03	0.833
Pulmonary artery diastolic pressure (mmHg)	19.3 ± 5.99	18.7 (15.7 – 22.0)	0.996	0.957 – 1.04	0.842
Pulmonary artery mean pressure (mmHg)	24.7 ± 6.87	23.8 (20.6 – 28.4)	1.00	0.958 – 1.05	0.955
Central venous pressure (CVP) (mmHg)	12.8 ± 4.17	11.9 ± 3.86	1.06	0.983 – 1.14	0.136
**Heart Rate (beats/min)**	**85.3 ± 23.6**	**91.6 ± 19.4**	**0.986**	**0.972 – 0.999**	**0.040**
**SpO_2 (%)**	**96.6 (94.4 – 97.9)**	**97.6 (96.6 – 98.8)**	**0.920**	**0.862 – 0.983**	**0.013**
Pulmonary artery pulsatility index (PAPi)	1.09 (0.833 – 1.47)	1.23 (0.944 – 1.67)	0.771	0.479 – 1.24	0.286
**Mean arterial pressure (MAP) (mmHg)**	**65.5 ± 7.20**	**69.8 (64.5 – 74.1)**	**0.918**	**0.878 – 0.961**	**<0.001**
Mean arterial pressure to central venous pressure ratio (MAP/CVP)	5.26 (4.30 – 6.48)	6.03 (4.67 – 7.72)	0.900	0.800 – 1.01	0.079

This table presents the pre-operative echocardiographic, blood test and pre-implant haemodynamic results for patients in the RVF and non-RVF groups. Pre-implant haemodynamic results are averaged over the last 6 hours prior to LVAD implantation. Non-parametric data are presented as counts and their corresponding percentages. Continuous data are presented as mean ± standard deviation if normally distributed, or median with interquartile range (IQR) for non-normally distributed data. Results of univariate logistic regression to identify variables that are significantly associated ($p<0.05$) with RVF following LVAD are presented. Their odds ratios, 95% confidence intervals and p-values are provided. Statistically significant results are bolded. N/A refers to variables where perfect prediction prevented these values from being obtained.

Table S3 – Post-hoc sensitivity analysis performed on candidate model

	AIC †	BIC ‡
Candidate model	144.6	162.5
Candidate model (+ ≥ pre-implant moderate tricuspid regurgitation)	143.7	164.5
Candidate model (+ pre-implant lymphocytes)	145.3	166.2
Candidate model (+ pre-implant heart rate)	146.5	167.3
Candidate model (+ pre-implant SpO_2)	144.2	165.1
Candidate model (+ pre-implant MAP)	144.1	164.9

This table presents the results of post-hoc sensitivity to evaluate the impact of potential confounders on the candidate model. Each row represents a new model where one potential confounder is added to the candidate model. The candidate model's AIC and BIC value were compared with each new model, with a drop in AIC or BIC of ≥2 indicating a better model fit.

† = Akaike Information Criterion; ‡ = Bayesian Information Criterion

Table S4 – Correlation matrix of variables in candidate model

Variables	CI	MAP	PPP	Pre-operative Inotropic Therapy
**Cardiac index (CI)**	1.0000			
**Mean arterial pressure (MAP)**	0.0457	1.0000		
**Pulmonary pulse pressure (PPP)**	0.3439	0.0452	1.0000	
**Pre-operative inotropic therapy**	-0.1250	-0.1009	-0.0598	1.0000

This table presents the pairwise Pearson correlation coefficients among the four variables in the candidate model, in order to assess for any potential multicollinearity. Each cell shows the correlation between the variables indicated by the respective row and column headers. Coefficients of >0.8 or < –0.8 were pre-determined as significant.

Table S5 – Performance metrics of the finalised model: overall results vs average results from 5-fold cross-validation with 5 seeds (at cut-off of p > 0.322)

Metric	Model Performance	Cross-Validation (Mean ± SD)
AUC	0.830	0.806 ± 0.0835
Accuracy	79.5%	70.4 ± 2.27 %
Sensitivity	76.7%	86.2 ± 3.09 %
Specificity	80.6%	63.9 ± 4.13 %
Positive Predictive Value (PPV)	62.3%	50.1 ± 2.30 %
Negative Predictive Value (NPV)	89.2%	91.8 ± 1.34 %

This table presents the predictive model's performance metrics at the chosen risk score cut-off of p>0.322. Results from both the overall analysis and the averages across a 5-fold cross-validation process with five different seeds are presented. The metrics considered include the Area Under the Curve (AUC), accuracy, sensitivity, specificity, Positive Predictive Value (PPV), and Negative Predictive Value (NPV). Each metric is listed with the corresponding values obtained from the final model and the means ± standard deviations from the cross-validation.

AUC: Indicates model's overall ability to correctly distinguish between those in RVF group from those in non-RVF group. Higher values suggest better performance.

Sensitivity: Indicates probability of correctly predicting patients who develop RVF

Specificity: Indicates probability of correctly predicting patients who do not develop RVF

Positive Predictive Value (PPV): Proportion of patients with positive test result who develop RVF.

Negative Predictive Value (NPV): Proportion of patients with negative test result who do not develop RVF.
